# Prophylactic Active Tau Immunization Leads to Sustained Reduction in Both Tau and Amyloid-β Pathologies in 3xTg Mice

**DOI:** 10.1038/s41598-017-17313-1

**Published:** 2017-12-06

**Authors:** Hameetha Rajamohamedsait, Suhail Rasool, Wajitha Rajamohamedsait, Yan Lin, Einar M. Sigurdsson

**Affiliations:** 1 Departments of Neuroscience and Physiology, New York University School of Medicine, 550 First Avenue, New York, NY 10016 United States; 2 Departments of Psychiatry, New York University School of Medicine, 550 First Avenue, New York, NY 10016 United States

## Abstract

Amyloid-β (Aβ) and tau pathologies are intertwined in Alzheimer’s disease, and various immunotherapies targeting these hallmarks are in clinical trials. To determine if tau pathology influences Aβ burden and to assess prophylactic benefits, 3xTg and wild-type mice received tau immunization from 2–6 months of age. The mice developed a high IgG titer that was maintained at 22 months of age. Pronounced tau and Aβ pathologies were primarily detected in the subiculum/CA1 region, which was therefore the focus of analysis. The therapy reduced histopathological tau aggregates by 70–74% overall (68% in males and 78–86% in females), compared to 3xTg controls. Likewise, western blot analysis revealed a 41% clearance of soluble tau (38–76% in males and 48% in females) and 42–47% clearance of insoluble tau (47–58% in males and 49% in females) in the immunized mice. Furthermore, Aβ burden was reduced by 84% overall (61% in males and 97% in females). These benefits were associated with reductions in microgliosis and microhemorrhages. In summary, prophylactic tau immunization not only prevents tau pathology but also Aβ deposition and related pathologies in a sustained manner, indicating that tau pathology can promote Aβ deposition, and that a short immunization regimen can have a long-lasting beneficial effect.

## Introduction

Alzheimer’s disease (AD) is characterized by accumulation of extracellular amyloid-β (Aβ) plaques, intracellular neurofibrillary tangles (NFT), and extensive synaptic loss leading to progressive cognitive impairment and eventually dementia. NFT are primarily composed of filaments of aggregated hyperphosphorylated tau protein^1^. Extensive work by numerous investigators suggests that Aβ pathology may lead to tau pathology^2^. However, it is interesting to note that reanalysis of a large number of human AD and control brains of various ages with phospho-specific tau antibodies revealed that phospho-tau immunoreactivity is generally detected in control brains prior to Aβ deposition^3^. This unexpected finding suggests that tau pathology may precede Aβ pathology in AD, at least in certain individuals, although it is of course unclear if these subjects would ever have developed the disease. Importantly, Aβ plaque clearance has had limited effect on tau pathology in the Aβ immunotherapy trials (for review see^4^), which emphasizes the need for therapy that specifically targets this other major hallmark of the disease. Numerous reports by us and others have shown the feasibility of tau immunotherapy^5–35^, and several clinical trials have been initiated (for review see^36^). However, all these studies were conducted in mice or related culture models that have only tau pathology but no Aβ pathology. Tau antibody immunization in Aβ plaque models has been reported to improve cognition and clear certain Aβ species while increasing Aβ plaque burden^37^, or when using a different tau antibody in a different Aβ model provided no cognitive benefits and increased mortality^38^. A few prior studies have reported toxic effects of active tau immunization in mice when very strong adjuvants are being used^39,40^.

One of the few models with both Aβ and tau deposits, is the triple transgenic (3xTg) mouse model, which harbors a presenilin 1 mutation (PS1/M146V) knock in allele, as well as the Swedish mutation of the amyloid precursor protein (APPSwe), and tau P301L mutation transgenes^41^. The PS1 and APP mutations individually cause familial AD and the tau mutation leads to frontotemporal dementia, which is an Aβ negative tauopathy. The 3xTg mice develop age-dependent and region-specific Aβ and tau deposits that mimic the disease progression in humans. They have previously received Aβ immunotherapy, which cleared Aβ plaques and rescued early but not late hyperphosphorylated tau aggregates^42^. More recently, a couple of studies have reported on the effect of tau antibodies on tau and Aβ burden in 3xTg mice^43,44^. A single injection of AT8, a phospho-tau antibody was shown to transiently reduce tau pathology without affecting Aβ pathology^43^. More recently, multiple injections of another tau antibody acutely reduced tau and Aβ pathology in their early stages^44^. Here, we report that immunization of this 3xTg model with Tau379–408[P-Ser396, 404] from 3–6 months of age, with animals killed at 22 months for analysis, resulted in a robust tau antibody response and long-term clearance of not only tau aggregates but also associated Aβ plaques.

## Materials and Methods

### Peptides

Phosphorylated tau peptide, Tau379–408[P-Ser396,404], was synthesized and purified at the Keck facility (Yale University) as described previously^5^.

### Transgenic Mice

The treatment was performed in 3xTg transgenic mice expressing knock-in mutation PS1/M146V combined with APP/K670N, M671L and MAPT/P301L transgenes^41^. A breeding pair of homozygous mice and another pair of wild-type (wt) mice of the same mixed strain background (C57BL6/129 SVJ) were graciously donated by Frank LaFerla (University of California at Irvine). These mice develop plaque and tangle pathology in AD relevant brain regions (hippocampus, cortex and amygdala). Wt mice from the same background were used as a control. The mice were housed in Association for Assessment and Accreditation of Laboratory Animal Care (AAALAC) approved facilities. All mouse care and experimental procedures were compliant with guidelines of animal experimentation and were approved by the Institutional Animal Care and Use committee at New York University School of Medicine.

### Vaccine Administration

At the start of the study, the 3xTg and wt mice were split into treatment groups that received the tau vaccine and control groups that received adjuvant alone (Table 1). The immunogen Tau 379–408 [P-Ser396, 404] was added to Adju-Phos adjuvant (Brenntag Biosector, Denmark) (1 mg/mL) and mixed overnight at 4 °C the day before injection for the peptide to adsorb onto the aluminum phosphate particles. The vaccine was injected subcutaneously (100 μl) with a second injection administered two weeks later and subsequent injections monthly thereafter. The treatment period was from 3-6 months of age (four injections). The mice were bled before the first immunization (T0) and then periodically thereafter to monitor their tau antibody response (T1: 1 week after the 3^rd^ immunization; T2-T5: 2, 5, 8 and 11 months after the 4^th^ immunization, respectively; Tf: 16 months after the 4^th^ immunization). At 22 months, their brains were extracted for analyses. The mice went through several behavioral tests in the two months prior to killing. At the end of study, a few mice had died in each of the four groups except in the immunized 3xTg groups, in which all the mice survived the experimental period (Table 1). Upon western blot analysis, 3 Tg mice (2 control males and 1 control female) were eliminated from the study because they did not express human tau, although the transgene was present. We have observed this at a similar rate in other Tg tauopathy models over the years. These three mice are not included in Table 1 or in any of the analyses.

**Table 1:** Number of mice of each gender at the beginning and end of the study.

	Start of Study	End of Study -Tissue Analysis
3xTg Control	19 (9 M + 10 F)	16 (6 M + 10 F)
3xTg Phos-tau	24 (13 M + 11 F)	24 (13 M + 11 F)
WT Control	14 (7 M + 7 F)	10 (4 M + 6 F)
WT Phos-tau	17 (8 M + 9 F)	15 (7 M + 8 F)

Only a few mice died during the study, and most of those were in the control group. None of the 3xTg treated mice died. M = male, F = female.

### Behavior

Each instrument was wiped clean with 30% ethanol between animals.

### Locomotor activity

A circular open field activity chamber (70 cm in diameter) was used to measure exploratory locomotor activity over 15 min^45^. A camera placed above the field recorded animal movements (San Diego Instruments). Measured parameters were distance traveled (in centimeters), mean resting time, and velocity [mean (*V*mean) and maximum (*V*max)] of the mouse.

### Traverse beam

This test measures balance, motor coordination and function integration^45^. Mice were evaluated by examining their ability to traverse a narrow beam to enter a goal box. The animals were placed on a wooden beam (1.1 cm wide, 50.8 cm long) that was suspended 30 cm above a soft foam cushion by two identical columns. At each end of the beam was attached a shaded goal box. Habituation consisted of placing the mouse on the middle of the beam for 60 s. Subsequently, in four successive trials, the number of foot slips before falling or reaching the goal box was recorded for each mouse. Errors were defined as foot slips and their numbers counted. A mouse that fell off the beam, was placed back on it at the location it fell from.

### Rotarod

This test was conducted to measure potential differences in forelimb and hindlimb motor coordination and balance without a practice confound (ref.^45^ Rotarod 7650 accelerating model; Ugo Basile). Habituation consisted of two trial training sessions to allow the animals to perform at a baseline level. Subsequently, the mice went through three test trials, with 15 min break between sessions. The rotarod was set 1.0 rpm and was gradually raised every 30 s until the mouse fell off or inverted (by clinging) from the rotating rod. The rpm at that point was recorded. To prevent injury, a soft foam cushion was beneath the rod.

### Cognitive Tests

Before each test, the mice were adapted to the room with lights on for 15 min.

### Radial arm maze

An eight–arm radial maze with a water well at the end of each arm was used to evaluate spatial learning^45^. Guillotine doors made from clear Plexiglas and operated by a remote pulley system, controlled access to the arms from the central circle from which the mouse entered and exited the maze. Following adaptation for 3–4 days, water-restricted mice (2 h daily access to water) went through one training session per day for ten consecutive days. In each session, all the arms of the maze were baited with saccharine flavored water, and the mouse was allowed to enter and explore all arms until the eight sugar rewards had been consumed. Spatial learning was assessed by recording the number of errors (entries to previously visited arms) and the time needed to complete each session.

### Closed field symmetrical maze

This maze consists of a rectangular field ( 63.5 cm square with 9 cm high walls divided into 36, 9.5 cm squares) that is covered by a clear Plexiglas top. Two boxes (each 15 × 20 × 9 cm), for the mice to enter or exit are situated at diagonal corners of the maze^45^. This symmetrical maze^46^ is based on the Hebb-Williams^47^ and Rabinovitch-Rosvold^48^ tests. Briefly, the key difference is that each end compartment serves as both a start box and a goal box, and the mouse navigates in opposite direction on alternate trials. An advantage of this setup is that it eliminates intertrial handling, which reduces animal stress and thereby gives more reliable data. The barriers are positioned symmetrically in the maze, so that the mouse faces the same turns going in either direction within a given setup. Before testing, the mice were adapted to a water restriction schedule, with 2 h daily access to water. Habituation consisted of two adaptation sessions before the first testing period. In the first such session, all the mice had access to saccharine flavored water in the goal box for 10 min. In the second adaptation session, the mouse was put in the start chamber and allowed to explore the field and enter the goal box, which contained saccharin water reward (0.05 mL). When the mice were consistently traveling between the start and goal boxes, they went through three practice sessions on simple navigational problems, in which one or two barriers were positioned in different regions of the maze to prevent direct route to the goal box. Formal testing consisted of three problems of varying difficulty, starting with the easiest one and ending with the most difficult one. The mice tackled one problem per day and the mice went through five trials to solve it with an inter-trial interval of 2 min. Their performance was scored by the same person for the number of errors (i.e. entries and reentries into designated error zones) and time to complete each trial.

### Antibody Response

The antibody response was determined in a 1:200 dilution of plasma using an ELISA assay as detailed previously^49,50^, in which the immunogen was coated on to microtiter wells (Immulon 2HB,Thermo Electron). The binding of plasma antibodies was detected by a goat anti-mouse IgG or IgM linked to a horseradish peroxidase (Pierce), with the enzyme catalyzing a color reaction in the substrate (tetramethyl benzidine; Pierce).

### Tissue Processing and Histology

After the behavioral testing, the mice were deeply anesthetized with ketamine/xylazine (250 mg/50 mg per kg body weight, i.p.). The brain was then extracted without perfusion and processed as detailed previously^51,52^. The left hemisphere was snap frozen on CO_2_ pellets and stored at −80 °C until processed for western blots. The right hemisphere was sectioned coronally (40 µm) from the frontal pole to the cerebellum, and sections were saved into 5 serial series for histological staining with about 40 sections per series. For each stain/antibody, at least half a series (20 sections), spaced equally apart (400 μm) were reacted. Staining was performed at room temperature as described previously^49,50,52^. Briefly, sections were placed in 0.3% H_2_O_2_ for 15 min to block endogenous peroxidase activity, and then incubated in mouse-on-mouse (MOM) blocking reagent (Vector Laboratories, Burlingame, CA) to block nonspecific binding for 1 h. Following washes in TBS-Tx, the sections were stained with PHF1 (1:2000 dilution of cell culture supernatant) and MC1 (1:100 dilution of cell culture supernatant) tau antibodies (generously provided by Dr. Peter Davies, Albert Einstein College of Medicine, Bronx, NY). Adjacent sections were also stained with 6E10/4G8 (1:2000 dilution of 1 mg/ml stock of each antibody) for Aβ deposits. To assess glial response, brain sections were stained with 1) rabbit polyclonal antibody against glial fibrillary acidic protein (GFAP) in astrocytes (1:500; Dako, Carpinteria, CA), and 2) Iba1 (10 µg/mL; Wako, Richmond, VA) to detect microglia. Neurons were stained with cresyl violet using standard procedure as described previously^53^.

For staining of microhemorrhages, serial coronal sections of experimental and control mouse brains were mounted on gelatin coated slides and stained with Prussian blue working solution as described previously^54^. Briefly, the brain sections were incubated in a mixture of equal volumes of 10% potassium ferrocyanide (K_4_Fe(CN)_6_ trihydrate) in distilled H_2_O and 20% hydrochloric acid (HCl) for 30 min. The sections were subsequently washed with H_2_O, counterstained with nuclear fast red solution for 10 min, washed again with H_2_O, dehydrated and coverslipped using Depex mounting media (BDH Laboratory Supplies, England).

### Image Analysis

Tau and Aβ deposition was analyzed in the subiculum of the brain because of its prominent and consistent such pathology. Tau pathology was quantified blindly in 5 sections per brain spaced 200 μm apart similar to as described previously^5,7,8^. The measurement was the percentage of area in the measurement field (200X) that was occupied by the reaction product (ImageJ, NIH). Aβ burden was analyzed as per our standard procedure^54^, using the StereoInvestigator Program (Area Fraction Fractionator; MBF Biosciences, Burlington, VT). The area of the grid was 800 μm^2^ × 800 μm^2^ and Aβ burden was measured in one frame per section (640 × 480 μm^2^ each) chosen randomly within the subiculum region, and five sections spaced 200 μm apart were analyzed. The Aβ burden is defined as the percentage of area in the measurement field (subiculum) that is occupied by the reaction product.

The assessment of the Iba1 (microglia) stained sections was based on a semi-quantitative analysis of microgliosis in the subiculum (0, predominantly resting microglia; 1+, a few ramified and/or phagocytic microglia; 2+, moderate number of ramified/phagocytic microglia; 3+, numerous ramified/phagocytic microglia). The rating of the GFAP sections was based on the complexity of astrocytic branching in the subiculum (1+, resting astrocytes, few processes; 2+, reactive astrocytes, moderate branching; 3+, reactive astrocytes, extensive branching).

The haemorrhage profiles (hemosiderin stain) were counted, and the average number of Prussian blue-positive deposits in the subiculum was calculated for each brain section.

All procedures were performed by an individual blinded to the experimental condition of the study. The accuracy of the findings was verified by two independent observers.

### Western Blotting

Brains were weighed and homogenized in modified RIPA buffer (50 mM Tris-HCl, pH 7.4, 150 mM NaCl, 1 mM EDTA, 1 mM NaF, 1 mM Na_3_VO_4_, 1 μg/ml complete protease inhibitor cocktail (Roche) and subjected to a low speed spin (14,000 rpm) to remove the membrane fraction. For sarkosyl extraction, 1% sarkosyl solution was added to 300 μL supernatant for a final concentration of 1% and then incubated for 1 h at 37 °C. Sarkosyl extracted supernatant and supernatant without sarkosyl were then centrifuged at 100,000 × *g* for 1 h at 4 °C in Beckman TL-100 ultracentrifuge, and the high-speed supernatants were collected and used for western blot analysis. Sarkosyl extraction results in dissociation of insoluble proteins including aggregated tau proteins. For the insoluble fraction, the pellet was re-suspended in the same volume of buffer without protease and phosphatase inhibitors, but that contained 1% (v/v) Triton X-100 and 0.25% (w/v) deoxycholate sodium. It was then ultracentrifuged at 50,000 × *g* for 30 min to retrieve a detergent extracted supernatant that was analyzed as an insoluble fraction^17,18^.

The supernatants from these three fractions were heated at 100 °C for 5 min and the same amount of protein was electrophoresed on a 12% (w/v) polyacrylamide gel. The proteins were then transferred to a nitrocellulose membrane that was subsequently blocked in 5% nonfat milk with 0.1% Tween-20 in TBS, and incubated with different antibodies for at least 3 h at room temperature or at 4 °C overnight (PHF1, CP27 generously provided by Peter Davies). Following washes, the membranes were then incubated for 2 h with 1:2000 horseradish-peroxidase (HRP) conjugated goat anti-mouse antibody (Pierce), developed in ECL (Pierce), imaged with Fuji LAS-4000, and the signal quantified with Multigauge.

### Experimental Design and Statistical Analyses

The experimental design was as detailed above. Briefly, transgenic and wt mice of both sexes received short-term prophylactic active tau vaccination at a young age (3–6 months). Control mice received only adjuvant. The mice were bled periodically to determine their antibody titers and they went through behavioral testing at the end of the study (20–22 months). Subsequently, their brains were removed for histological and biochemical analyses of the effects of the vaccination on AD related pathologies, and to monitor potential adverse effects.

All data were analyzed with GraphPad Prism 7. Unless specified below, the analysis was performed with an unpaired t-test, two-tailed. Welch correction was used if the data failed a test of equal variance. When the data failed at least two of three normality tests (Kolmogorov–Smirnov, D’Agostino and Pearson omnibus, and Shapiro–Wilk normality tests), nonparametric Mann–Whitney test was used. That test was also used for analyzing the astro- and microgliosis. Behavioral data was analyzed with one or two-way ANOVA, depending on the number of factors. When the data failed at least two of three normality tests (Kolmogorov–Smirnov, D’Agostino and Pearson omnibus, and Shapiro–Wilk normality tests), nonparametric Kruskal Wallis test was used. Radial arm maze test was analyzed with two-way ANOVA, repeated measures.

## Results

### Tau immunization elicits a robust antibody response

**Figure 1:**
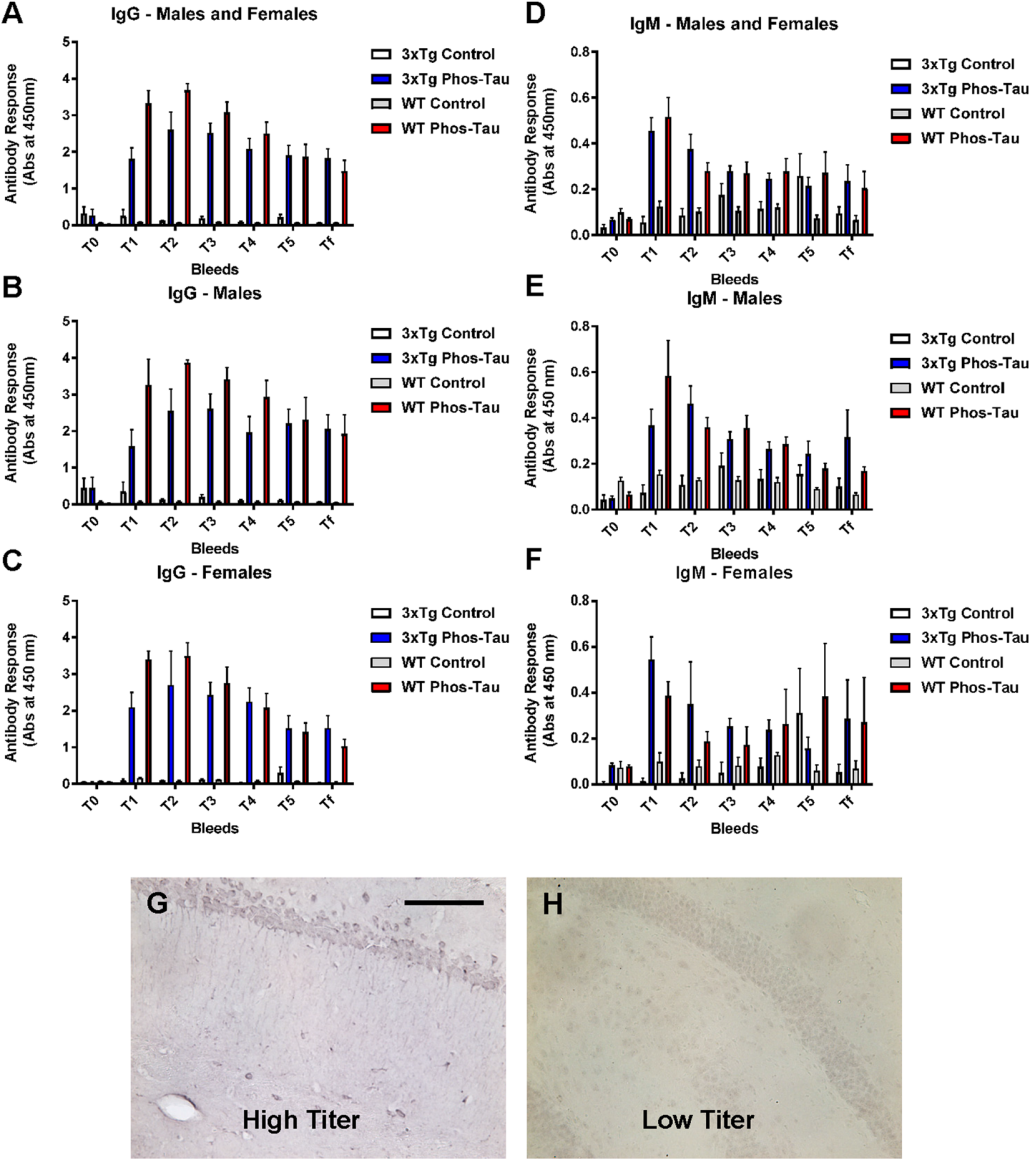
The tau immunogen Tau379–408[P-Ser396, 404] elicits a robust and sustained antibody response. (**A**–**C**) IgG response towards the immunogen was strong and long-lasting, and comparable in males vs. females. It was strong at T1 (1 week after the 3^rd^ immunization), peaked at T2 (2 months after the 4^th^ and last immunization) and remained strong thereafter (T3-Tf: 5, 8, 11, and 16 months after the 4^th^ immunization). Higher IgG levels were detected in wt mice compared to 3xTg mice. (**D**–**F**) IgM response was not as strong as the IgG response but showed a similar pattern as the IgG response except that the IgM response was comparable in the wt vs. the 3xTg mice. (**G**,**H**) Plasma (1:100) obtained from a high titer mouse at the end of the study (Tf) stained tau aggregates in a control 3xTg mouse, whereas plasma from a low titer control mouse did not. Scale bar: 125 μm.

 Mice immunized with the Tau 379–408[P-Ser396, 404] immunogen in Adju-Phos adjuvant developed a robust IgG response in both male and female 3xTg as well as wt mice compared to controls that received adjuvant alone. (Fig. 1A–C). IgM response was less pronounced in the same groups (Fig. 1D–F). Notably, the mice maintained high antibody levels, after the fourth and last immunization at 6 months of age, until the end of the study when the mice were 22 months of age. This was evident both in Tg and wt mice. Plasma from a high titer mouse stained intraneuronal tau aggregates, whereas plasma from a control mouse did not (Fig. 1G,H), which confirms our previous findings that polyclonal antibodies elicited to this vaccine recognize pathological tau protein^5,10^. In a pilot study, we noticed substantial mortality in the immunized mice after the 5^th^ immunization, with surviving mice maintaining high antibody titer for more than a year. Hence, the enrolled mice received only four vaccine injections. That short vaccination paradigm did not appear to elicit side effects. As noted in Table 1, only a few mice died in three of the groups and none in the immunized 3xTg mice. Based on prior work with this vaccine in other mouse models, the mortality in the pilot study is likely related to more robust immune response to this vaccine in mice on this particular mixed strain background.

### Cognitive benefits cannot be detected with the immunotherapy as the Tg mice are not impaired compared to wt mice

The tau immunotherapy did not lead to any significant cognitive improvement in the immunized 3xTg mice, compared to adjuvant treated controls (data not shown). Our analysis however, did not detect cognitive impairments in the 3xTg controls compared to wt controls. Hence no therapeutic behavioral benefits were observed with the immunotherapy as the mice were not impaired. We have used these same tests extensively in other tangle models^5,7,8,34^, and observed that Tg tau immunized mice performed better than the Tg control mice. These prior studies include the use of the same tau immunogen. The immunized 3xTg mice and wt mice did not differ significantly from their non-immunized identical control mice in any of the cognitive tasks, and all the groups appeared to have normal motor functions based on our experience with wt mice in these tests (data not shown).

### Tau immunization decreases tau pathology

**Figure 2:**
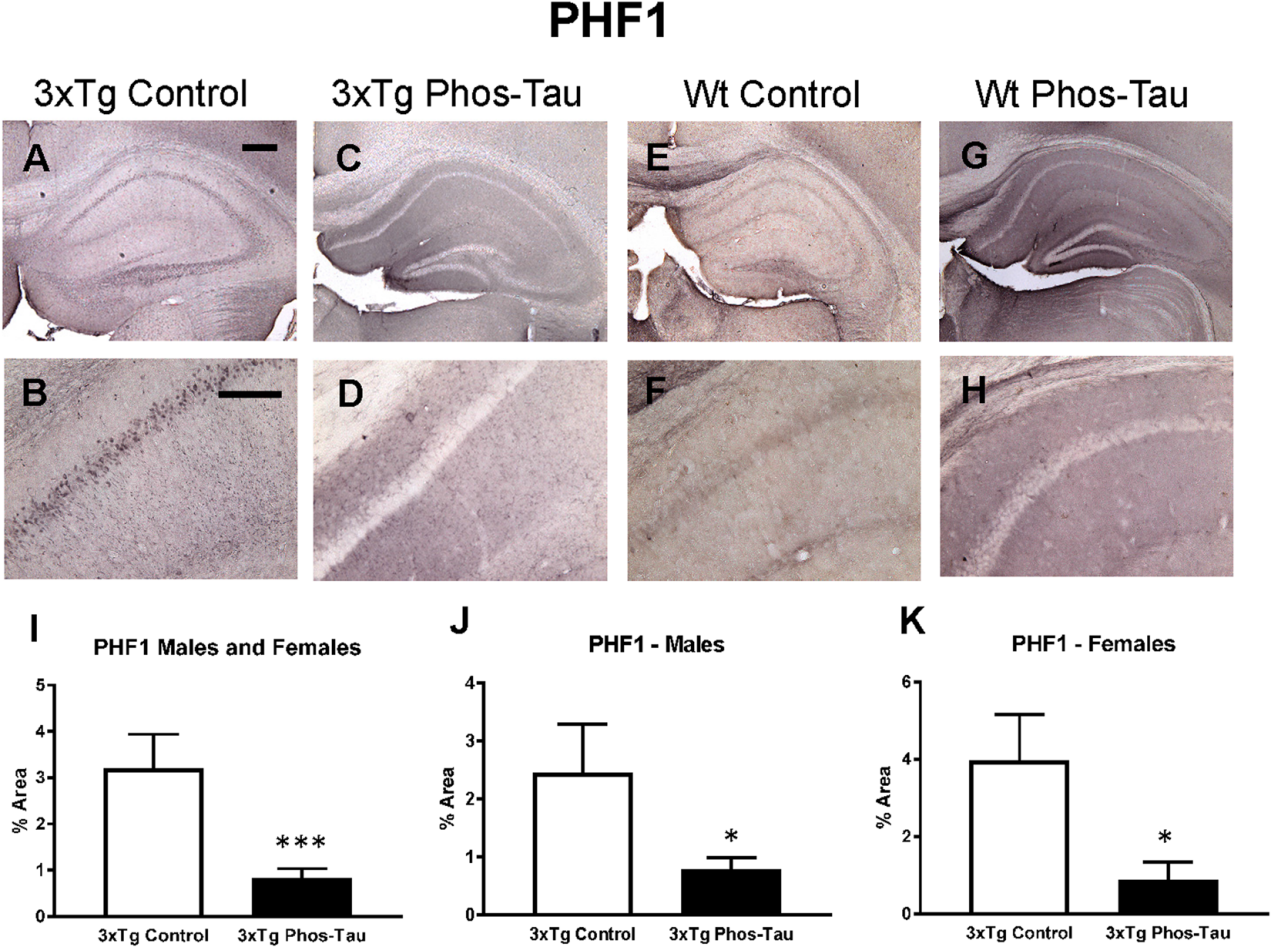
Tau immunization prevents phospho-tau histopathology. (**A**–**D**) PHF1 immunostaining revealed extensive tau pathology in the hippocampus (A: 5X objective, B: 20X objective) of 3xTg mice, that was greatly reduced in immunized mice (**C**,**D**). (**E**–**H**) Only background staining was seen in the wt mice. (**I**–**K**) Quantitative analysis of the PHF1 staining revealed significant reduction in tau aggregates in the combined group (**I**: 74%, p = 0.0008) as well as in males (**J**: 68%, p = 0.0437) and females (**K**: 78%, p = 0.0120) analyzed separately. Scale bar: 250 μm (**A**), 125 µm (**B**). *p < 0.05; ***p < 0.001 compared to 3xTg control. See text for exact p-values.

**Figure 3:**
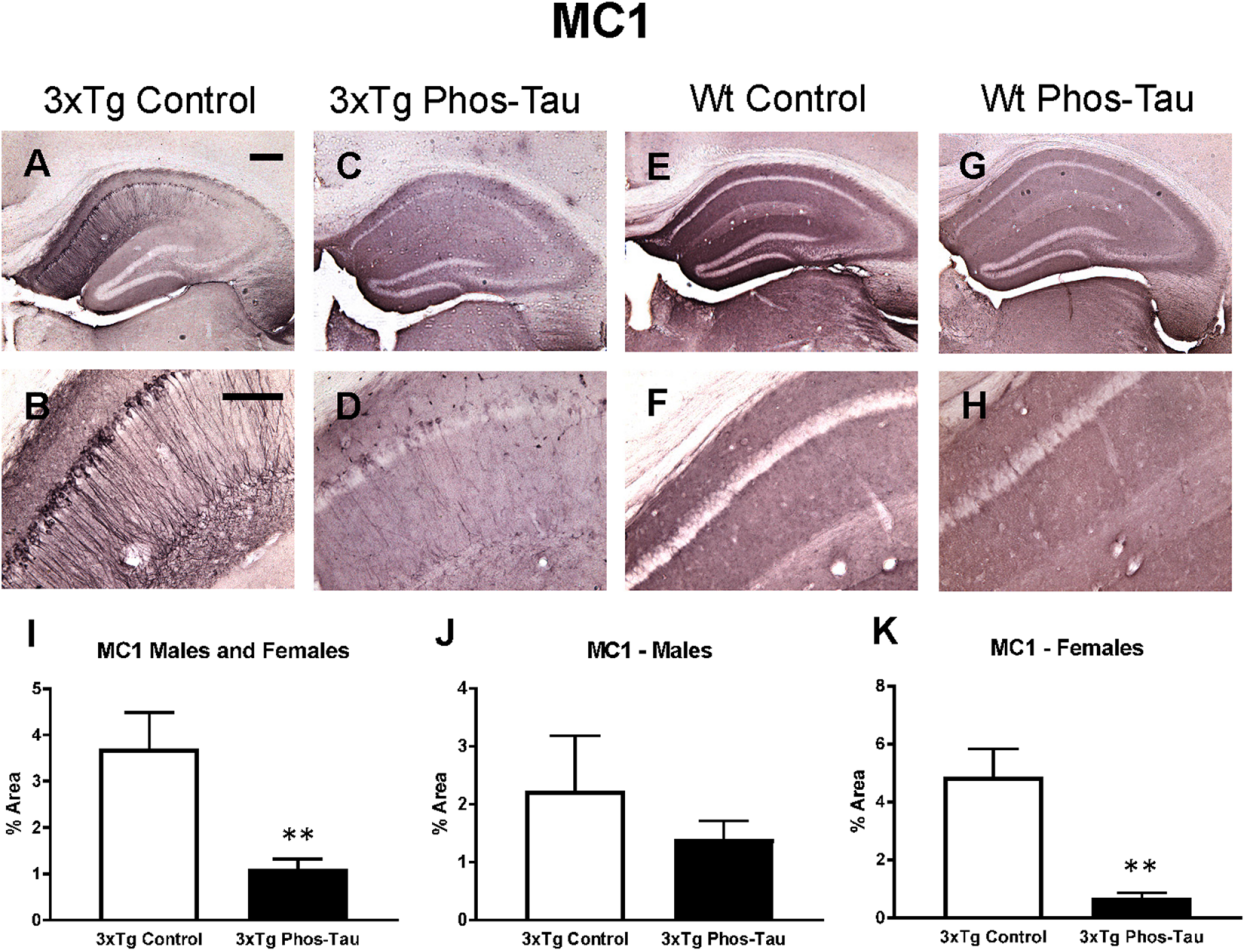
Tau immunization prevents conformational tau histopathology. (**A**–**D**) MC1 immunostaining revealed extensive tau pathology in the hippocampus (A: 5X objective, B: 20X objective) of 3xTg mice, that was greatly decreased in immunized 3xTg mice (C-D). (**E**–**H**) Only background staining was seen in the wt mice. (**I**–**K**) Quantitative analysis of the MC1 staining revealed significant reduction in tau aggregates in the combined group (I: 70%, p = 0.0057) as well as in females (**K**: 86%, p = 0.0070). Scale bar: 250 μm (**A**), 125 µm (**B**). **p < 0.01 compared to 3xTg control. See text for exact p-values.

To determine the consequences of the active tau immunization on hyperphosphorylated tau and pathological tau conformers, brain sections were stained with PHF1 and MC1 antibody, respectively. Pronounced tau pathology was revealed with both antibodies, primarily in the subiculum/CA1 region, which was therefore the focus of analysis (Figs 2 and 3A–D). Only background staining was seen in the wt mice (Figs 2 and 3E–H). It may relate to their old age and because the mice were not perfused. The therapy reduced PHF1-reactive tau aggregates by 74% in the combined 3xTg male and female group (Fig. 2I, p = 0.0008), which was also significantly reduced in males (Fig. 2J; 68%; p = 0.0437) and females (Fig. 2K, 78%; p = 0.0120), compared to identical controls. MC1 immunoreactive tau aggregates were also reduced in the immunized 3xTg male and female combined group (Fig. 3I, 70%, p = 0.0057), and in the separate female group (Fig. 3K, 86%; p = 0.0070), compared to identical controls.

**Figure 4:**
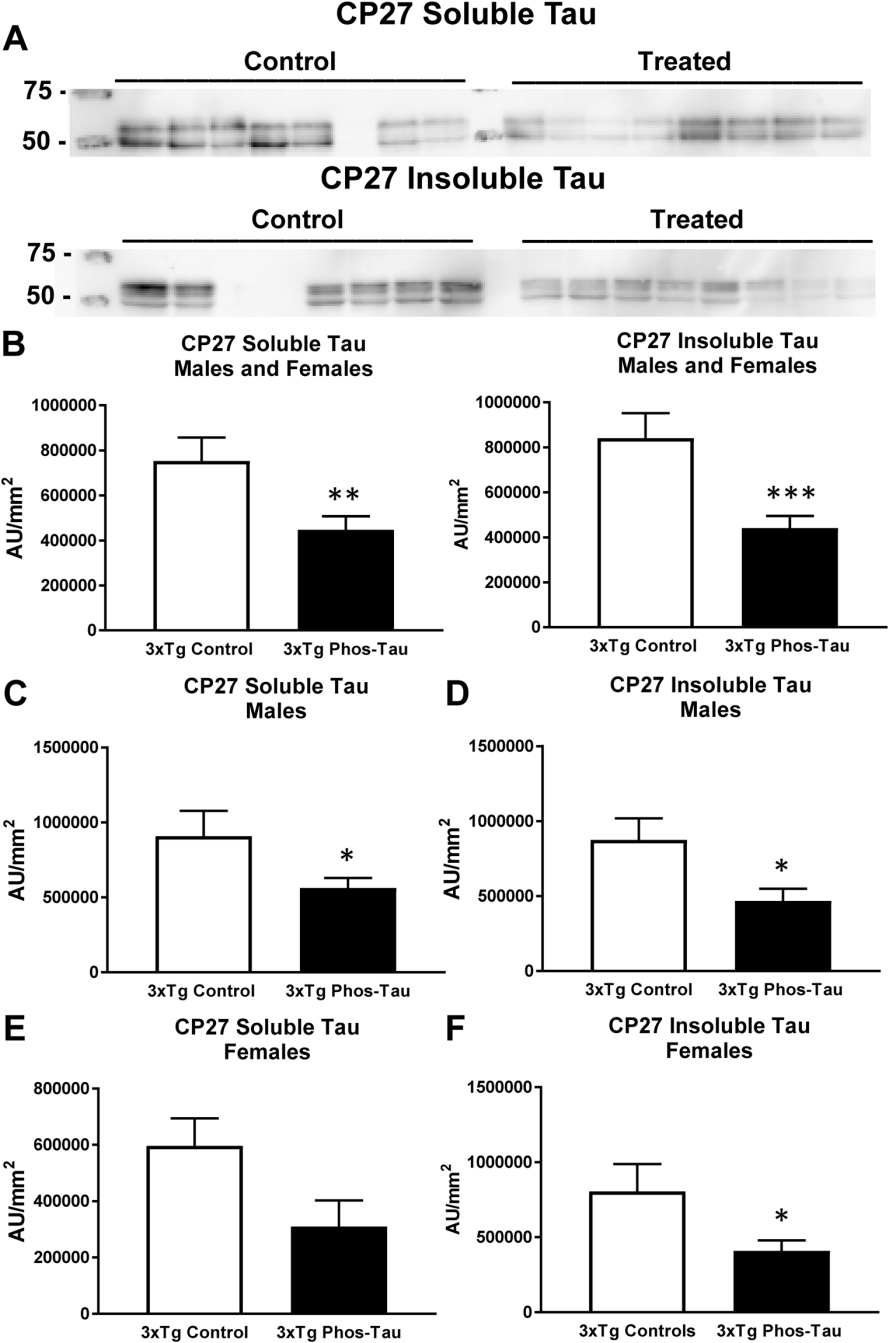
Tau immunization decreases soluble and insoluble human tau protein. (**A**,**B**) Representative blots are shown in A. Note that lanes that appear empty are from mice that did not express human tau and were, therefore, omitted from all analysis. Western blot analysis revealed that soluble and insoluble CP27-reactive human tau were reduced in the immunized 3xTg mice (soluble tau: 41%, p = 0.0103; insoluble tau: 47%, p = 0.0008). (**C**–**F**) Comparable tau reductions were observed in males (soluble tau: 38%, p = 0.0322; insoluble tau: 47%, p = 0.0165) vs. females (soluble tau: 48%, p = 0.0616; insoluble tau: 49%, p = 0.0477). *p < 0.05; **p < 0.01; ***p < 0.001 compared to 3xTg control. See text for exact p-values.

**Figure 5:**
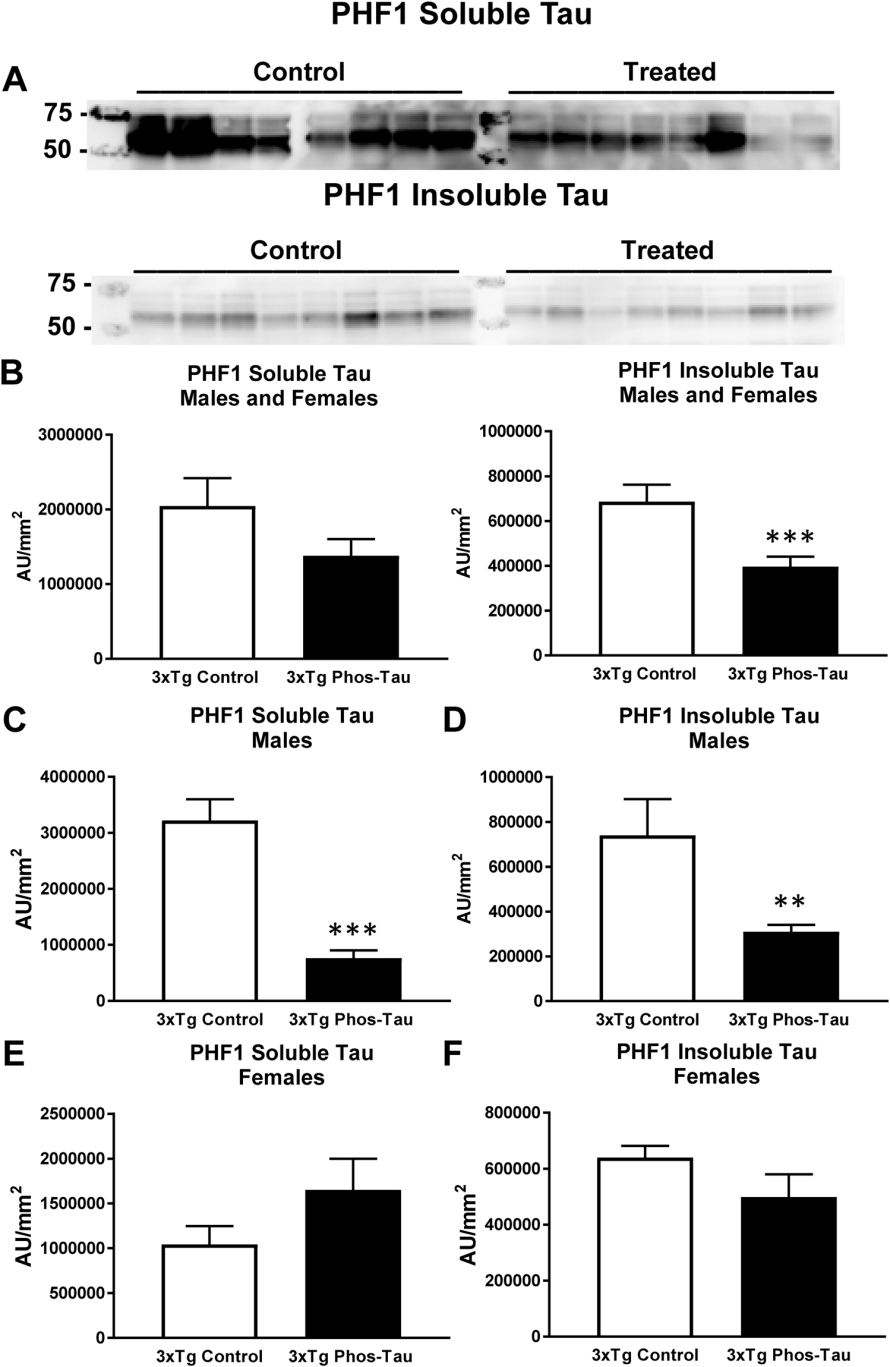
Tau immunization reduces soluble and insoluble phospho-tau protein. (**A**,**B**) Representative blots are shown in **A**. Western blot analysis revealed that insoluble PHF1-reactive human tau was decreased in the immunized 3xTg mice (42%, p = 0.0003). (**C**,**D**) Both soluble (76%, p = 0.0001) and insoluble tau (58%, p = 0.0018) were decreased in the males but not in the females (**E**,**F**). **p < 0.01; ***p < 0.001 compared to 3xTg control. See text for exact p-values.

Likewise, western blot analysis revealed a similar clearance of tau in the immunized 3xTg mice. Significant reductions were observed in soluble and insoluble human tau (CP27) in the immunized overall group (Fig. 4A,B, soluble tau: 41%, p = 0.0103; insoluble tau: 47%, p = 0.0008), and in males (Fig. 4C,D, soluble tau: 38%, p = 0.0322; insoluble tau: 47%, p = 0.0165), and females (Fig. 4E,F, soluble tau: 48%, p = 0.0616; insoluble tau: 49%, p = 0.0477), analyzed separately compared to Tg controls. For PHF1 reactive phospho-tau, significant reductions were detected in the insoluble tau fraction in the immunized combined group (Fig. 5B, 42%, p = 0.0003), and in males in soluble (Fig. 5C, 76%, p = 0.0001) and insoluble tau (Fig. 5D, 58%, p = 0.0018). The immunization did not affect endogenous tau in the wt mice (Tau-5: wt controls = 456,740 ± 76,011 AUC/mm^2^ (average ± SEM); wt immunized = 376,469 ± 98,775 AUC/mm^2^).

### Tau immunization reduces Aβ plaque burden

**Figure 6:**
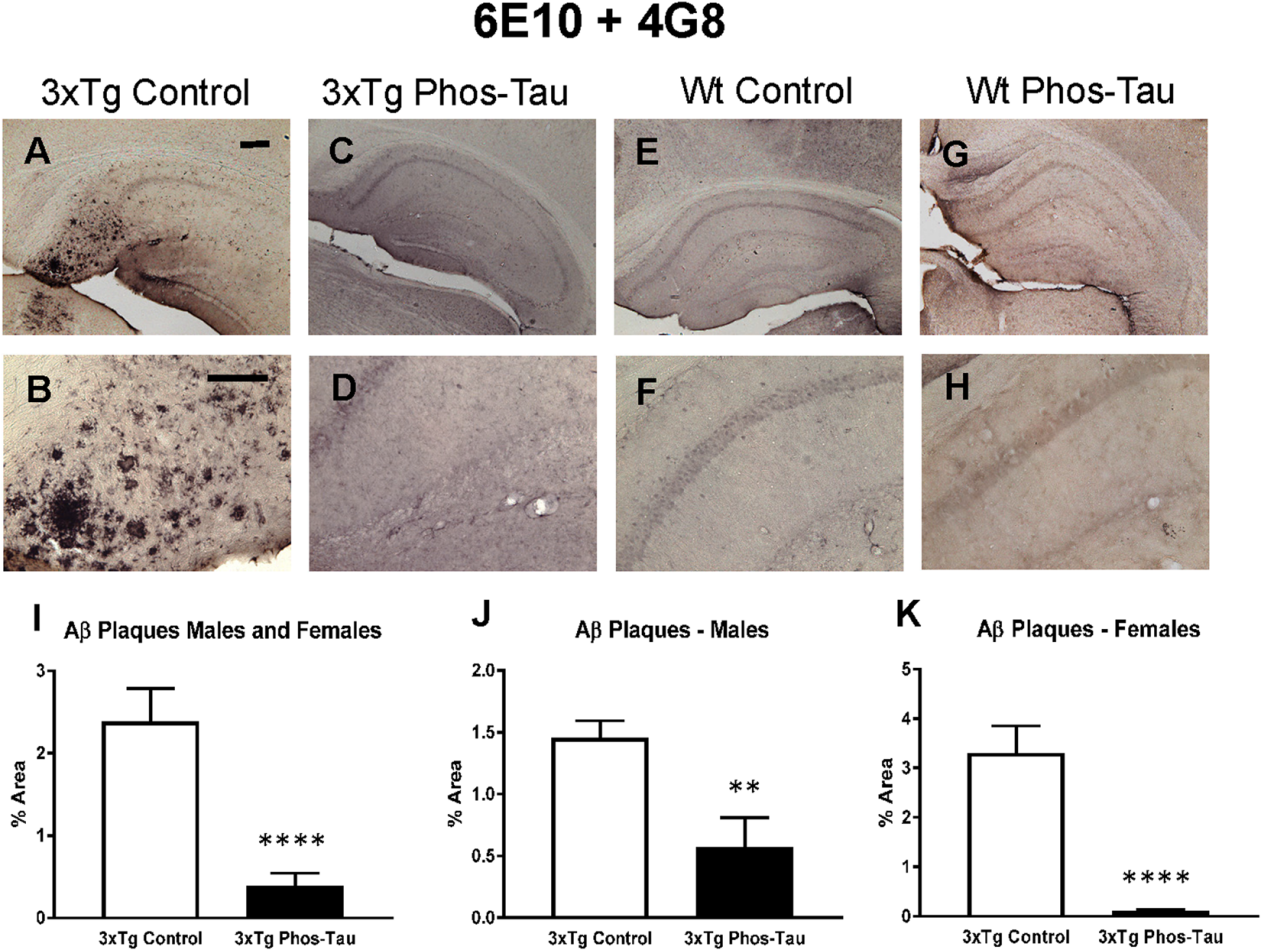
Tau immunization diminishes Aβ burden. (**A**–**D**) Aβ immunostaining revealed extensive Aβ plaque burden in subiculum region of the hippocampus (**A**: 5X objective, **B**: 20X objective) of 3xTg mice, that was greatly reduced in immunized mice (**C**,**D**). (**E**–**H**) Only background staining was seen in the wt mice. (**I**–**K**) Quantitative analysis of the Aβ staining revealed significant reduction in Aβ plaque burden in the combined group (**I**: 84%, p < 0.0001) as well as in males (**J**: 61%, p = 0.0033) and females (**K**: 97%, p = 0.0001) analyzed separately. Scale bar: 250 μm (**A**), 125 µm (**B**). **p < 0.01; ****p < 0.0001 compared to 3xTg control. See text for exact p-values.

Aβ plaque burden in the subiculum region of the 3xTg mice was analyzed by immunohistochemistry using a combination of 6E10 and 4G8 antibodies (Fig. 6A–D). Only background staining was seen in the wt mice (Fig. 6E–H). Aβ deposits in tau immunized 3xTg mice were decreased significantly as compared to control vaccinated mice (Fig. 6I–K, 84% in combined group, p < 0.0001; 61% in males, p = 0.0033, and; 97% in females, p < 0.0001). These results indicate that prophylactic tau immunization reduces the formation of Aβ plaque deposits.

### Tau immunization does not appear to affect neuronal density

Like many other AD models, 3xTg mice do not have extensive neuronal loss^41,55^. We stained brain sections with cresyl violet from Tg mice that had a robust therapeutic response to the vaccine, and compared those to sections from adjuvant control 3xTg mice. Neuronal density/numbers appeared to be similar in these two groups (data not shown), which fits with the previously reported limited effect of tau or Aβ pathology on this parameter in this model^41,55^.

### Tau immunization reduces microgliosis and microbleeds but does not affect astrocytes

**Figure 7:**
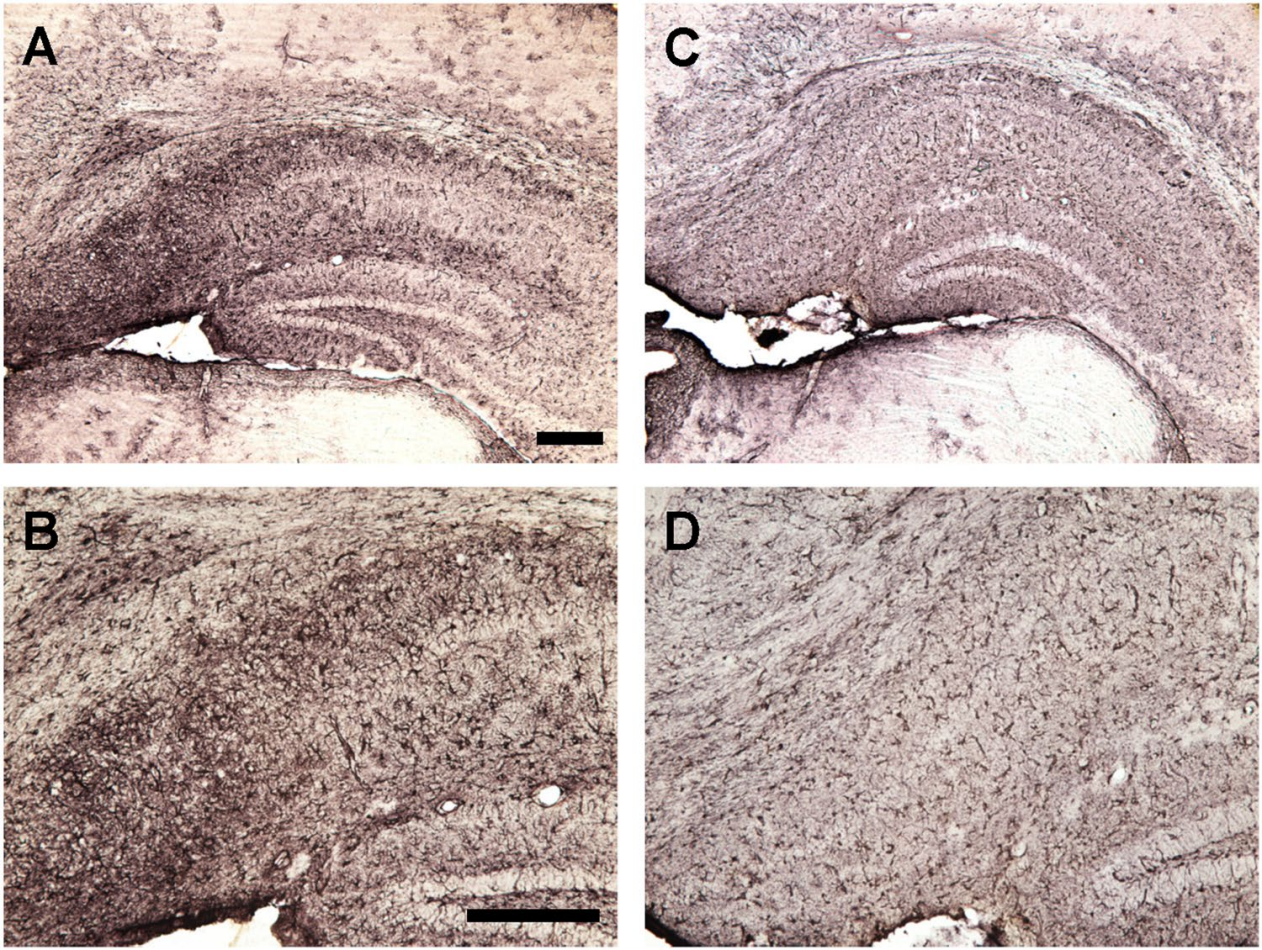
3xTg mice have more astrogliosis than wt mice. (**A**–**D**) GFAP immunostaining revealed extensive astrogliosis in subiculum region of the hippocampus (**A**: 5X objective, **B**: 10X objective) of 3xTg mice, that was much less in WT mice (**C**,**D**). Scale bar: 250 μm.

**Figure 8:**
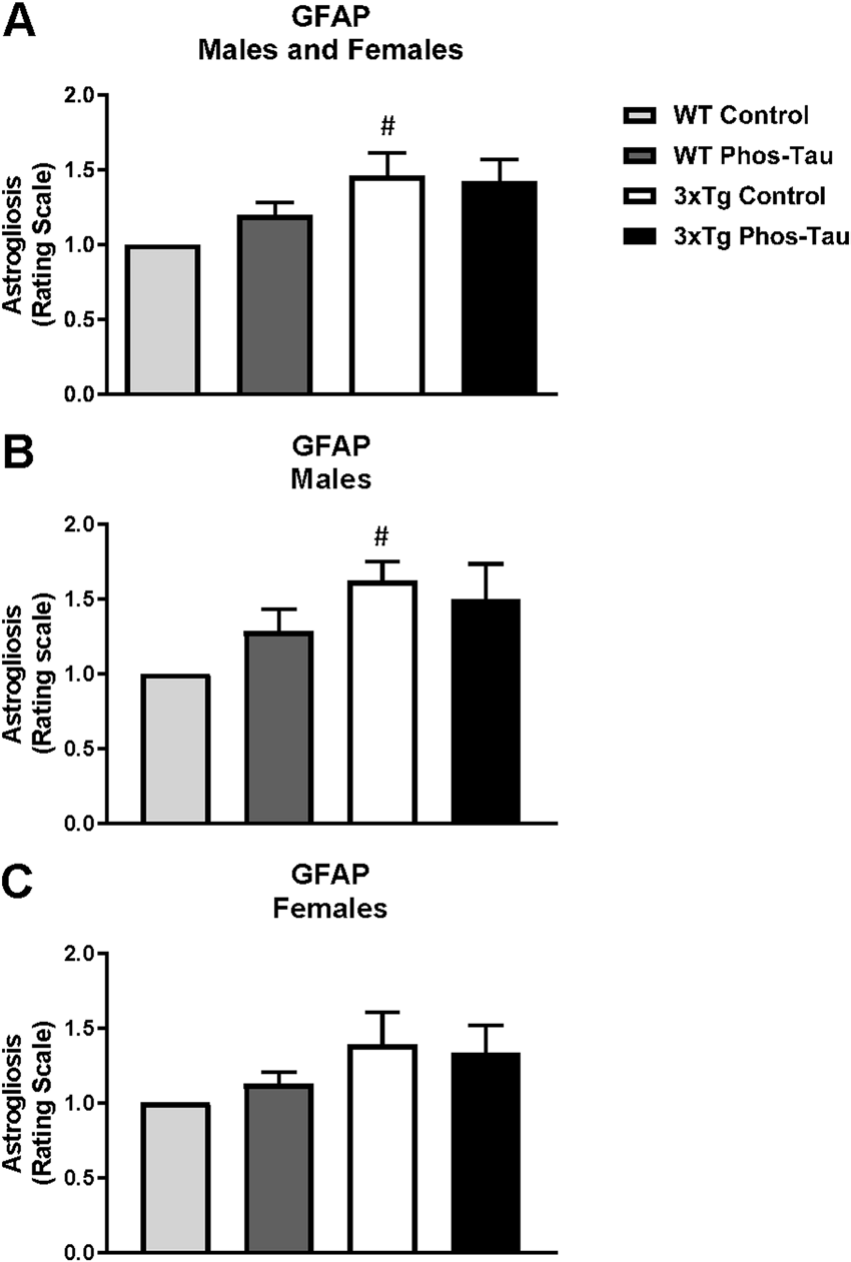
3xTg mice have more astrogliosis than wt mice. (**A**–**C**) Semi-quantitative analysis of GFAP immunoreactivity in the subiculum revealed that 3xTg mice had more astrogliosis than wt mice (p = 0.0139) that was also significantly increased in the males (p = 0.0286). However, astrogliosis was not significantly affected by the tau immunotherapy in the combined group (**A**) or in the males (**B**) and females (**C**) analyzed separately. ^#^p < 0.05 compared to wt control. See text for exact p-values.

**Figure 9:**
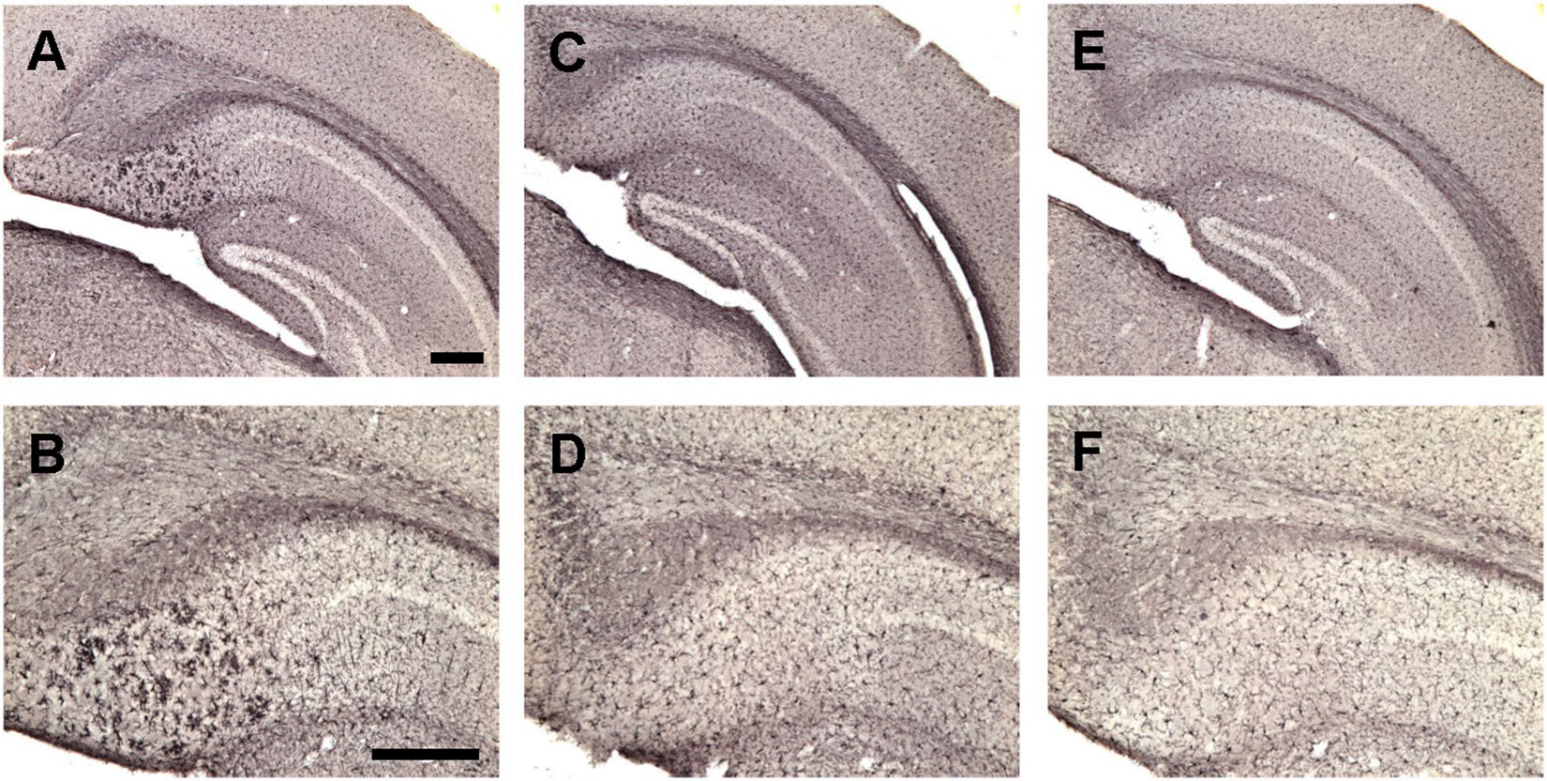
Tau immunization reduces microgliosis in 3xTg mice to wt levels. (**A**–**F**) Iba1 immunostaining revealed extensive microgliosis in the subiculum region of the hippocampus in 3xTg mice (**A**: 5X objective; **B**: 10X objective) that was greatly reduced in tau immunized mice (**C**,**D**) rendering these animals indistinguishable from wt mice (**E**,**F**). Scale bar: 250 μm.

**Figure 10:**
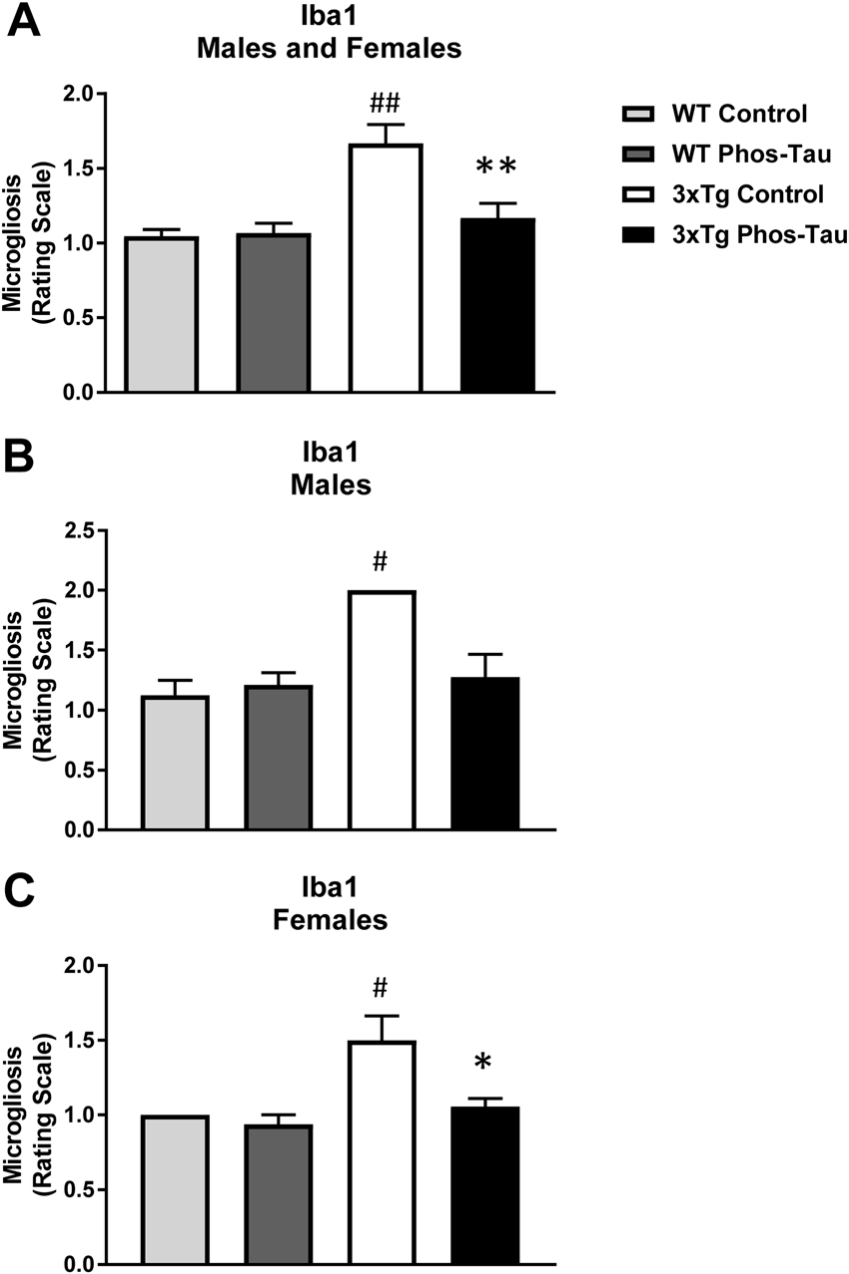
Tau immunization reduces microgliosis in 3xTg mice to wt levels. (**A**–**C**) Semi-quantitative analysis of Iba1 immunoreactivity in the subiculum revealed that 3xTg mice had more microgliosis than wt mice (males and females: p = 0.0013; males: p = 0.0286; females: p = 0.0310), and the tau immunotherapy reduced microgliosis in 3xTg mice to wt levels (males and females: p = 0.0056; males: p = 0.0699; females: p = 0.0294). ^#^p < 0.05; ^##^p < 0.01 compared to wt control. *p < 0.05; **p < 0.01 compared to 3xTg control. See text for exact p-values.

**Figure 11:**
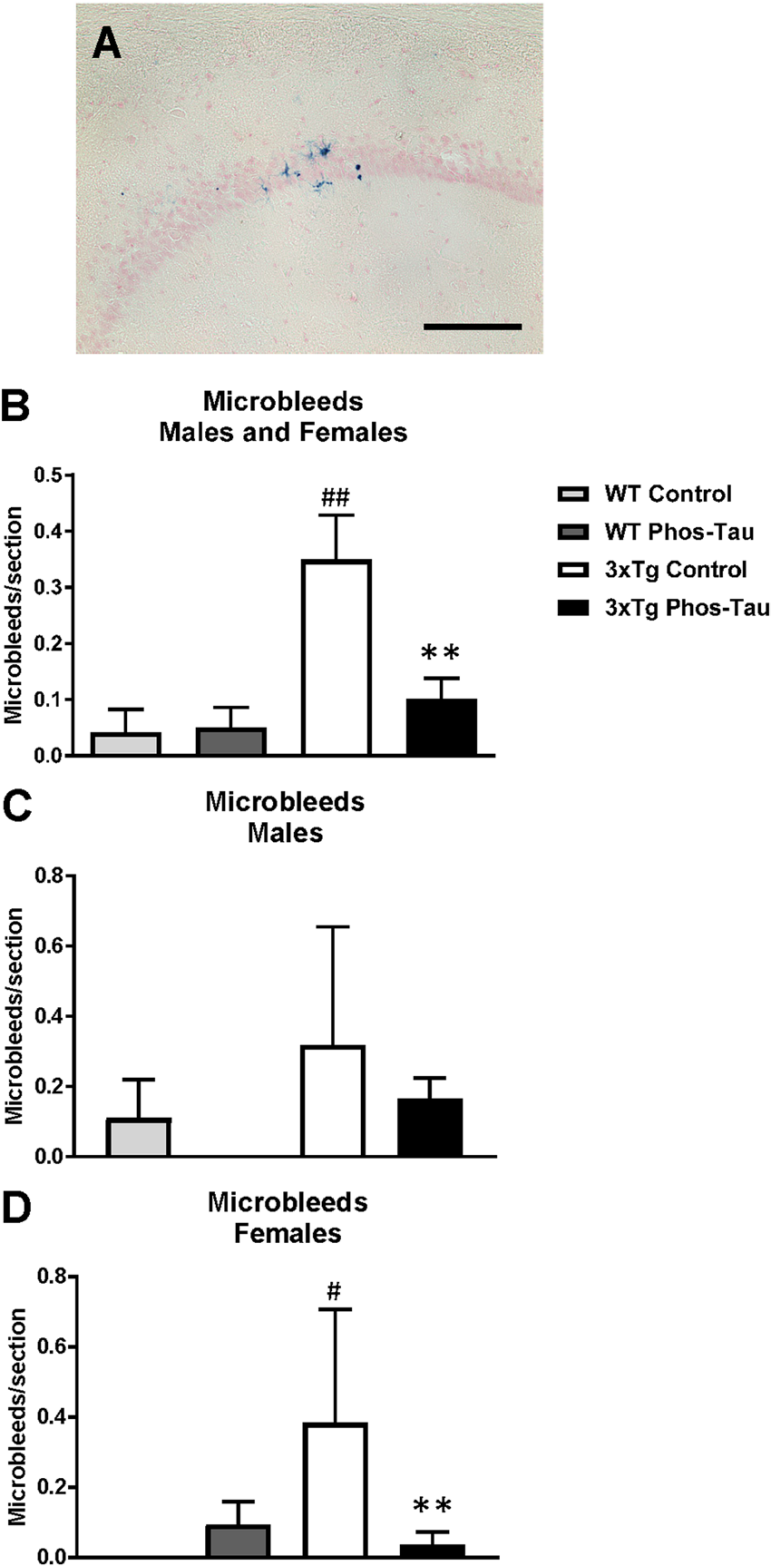
Tau immunization diminishes microbleeds in 3xTg mice to wt levels. (**A**–**D**) A few microbleeds (blue) were primarily detected in the hippocampus (**A**: 20X objective) of the 3xTg control mice (males and females: p = 0.0088 compared to wt control; females: p = 0.0165), and their numbers were significantly reduced in the tau immunized 3xTg mice (males and females: p = 0.0095; females: p = 0.0078) to wt levels. Scale bar:  125 μm. ^#^p < 0.05; ^##^p < 0.01 compared to wt control. **p < 0.01 compared to 3xTg control. See text for exact p-values.

To assess the potential involvement of activated astrocytes, microglia and microhemorrhage following immunization, histological analysis was performed focusing on the subiculum of the hippocampus, the region with the highest Aβ plaque burden and associated tauopathy. GFAP immunoreactivity was greater in 3xTg mice compared to wt mice (males and females: p = 0.0139; males: p = 0.0286; females: 0.2286) but the immunotherapy did not affect astrogliosis (Figs 7 and 8). Likewise, microgliosis was more pronounced in 3xTg mice than in wt mice (males and females: p = 0.0013; males: p = 0.0699; females: 0.0294) but the immunotherapy reduced it significantly in the Tg mice (males and females: p = 0.0056; males: p = 0.0699; females: p = 0.0294, Figs 9 and 10). For microhemorrhages, those were also seen more often in 3xTg mice compared to wt mice (males and females: p = 0.0088; males: p = 0.5714; females: p = 0.0165), and were reduced significantly in the treated Tg mice compared to their Tg controls (males and females: p = 0.0113; males: p = 0.4725; females: p = 0.0078, Fig. 11). These results suggest that gradual removal of tau aggregates, and an indirect clearance of Aβ deposits, are not associated with extensive gliosis or microhemorrhages, and actually reduce microgliosis and microhemorrhages. This lack of treatment associated gliosis is in accordance with our prior results with this immunogen or tau antibody in other tauopathy models^5,7,8^. Overall, these results support the view that Aβ and tau pathologies are synergistic. Clearing tau leads indirectly to clearance of Aβ and associated pathologies such as microgliosis and microhemorrhages.

## Discussion

Our findings indicate that tau immunotherapy can not only lead to clearance of tau pathology but also of Aβ deposits. Importantly as well, the benefits are sustained, lasting at least 16 months following the last immunization. Together, these findings may have major implications for clinical use of this approach. Aβ immunotherapy has previously been shown to reduce tau pathology to a modest extent in mouse models and humans (reviewed in^4^). Also, a single intrahippocampal injection of a tau antibody reduces early and late tau pathology in 3xTg mice without affecting Aβ deposits^43^. In that particular study, clearance of tau pathology was acute and transient, observed at 7 and 14 days post-injection but was no longer evident 21 days after antibody administration. It is, therefore, not surprising that Aβ pathology was not affected within such a short timeframe. More recently, tau passive immunization was shown to inhibit not only tau but also Aβ pathology in 3xTg mice that received 6 weekly tau antibody injections at the early stages (12 months) of tau pathology in this model^44^. In our study, the mice received their first vaccine injection at 3 months, and the last one at 6 months, at which age the mice have minimal if any tau or Aβ pathology. The tau antibodies elicited by the vaccine then prophylactically prevented the development of intraneuronal tau aggregates, which then indirectly attenuated Aβ deposition. Further support for the synergistic effects of these two pathologies can be obtained from their regional colocalization in the 3xTg model. The Aβ deposits are most prominent in the subiculum region of the hippocampus and are surrounded by dystrophic neurites positive for pathological tau protein. It can be inferred that neurons with tau lesions generate more Aβ than healthy neurons that is then deposited near their synaptic terminals. This scenario would then further promote tau pathology and enhanced Aβ deposition resulting in a vicious cycle. Long-term antibody-mediated removal of tau aggregates would therefore be expected to attenuate the progression of the intertwined tau and Aβ pathologies as confirmed by our findings.

The tau immunotherapy reduced tau and Aβ burden in both sexes but the therapeutic benefits were generally more pronounced in females, although they have more pronounced pathology, which fits our prior findings in a different tauopathy model^7^. Associated pathology, microgliosis and microbleeds were also reduced more significantly in females than males following the immunotherapy, which would be expected as these are closely linked to Aβ deposits.

Cognitive impairment has been previously documented in 3xTg mice (for review see^56^). We were not able to detect such impairments in two different cognitive tests compared to age-matched littermate wt mice. The performance of the wt controls in this study was comparable to our prior reports using these tasks in wt controls of a different strain background but of a similar age^50,54^. This further supports lack of cognitive deficits of the 3xTg mice in these tasks. However, studies reporting memory issues in this model used different tests. Also, the 3xTg mice we used may have had less severe pathology than in reported studies. Finally, the mice in our study were older than in the prior report. It is conceivable that age-related memory deficits in wt mice may catch up with Tg deficits at the 21–22 months of age when our mice were tested. We are not aware of other reports assessing cognition in this model in mice older than 15–18 months of age^56^. Most of these studies report deficits at various ages ranging from 3–5 months to 15–18 months with a few showing no impairment compared to controls at 1–2 months, 3–5 months and 9–11 months. None of the tests in the prior studies were similar to ours, which further complicates comparison.

Regardless of the lack of a pronounced behavioral phenotype in the 3xTg mice compared to wt mice of the same strain background, the histological effects of the tau immunotherapy were pronounced and highly significant. These findings suggest that prevention of the development of tau pathology can robustly diminish associated Aβ deposition in mice that are prone to develop such deposits because of APP and PS1 mutations. Remarkably, the therapeutic benefits of the prophylactic immunization were long-lasting, up to 16 months after the fourth and last immunization. Prior active tau immunization studies have not assessed such sustained benefits following the vaccination paradigm. Typically, the final immunization in those studies has been within a month prior to brain analysis. What led us down the path of determining possible long-term benefits of active tau immunization was the unexpected high mortality in this model following the fifth immunization in a pilot study that preceded this comprehensive study. Such catastrophic adverse effects most likely relate to the strong immune response in this particular hybrid strain because 3xTg and wt mice on the same strain background were affected to a similar extent. In our prior studies using this tau immunogen and the same alum adjuvant, we did not observe adverse reactions let alone death in two different tauopathy mouse models that received five or more immunizations^5,7^. Here, the four immunizations did not appear to lead to side effects. All the 3xTg immunized mice survived until the end of the study, and in each of the other three groups (3xTg and wt controls as well as wt immunized) only a few mice died during the long experimental period (Table 1).

The immunization did not affect tau levels in wt mice. This is as expected because of the phospho-tau immunogen whose epitope is primarily found in pathological tau. Also, the normal tau in wt mice is primarily cytosolic, whereas tau antibodies within neurons typically bind to pathological tau in endosomal-lysosomal vesicles ^10,17,18,21,34,57^.

It remains to be seen if such prolonged prophylactic benefits, not only for blocking tau pathology but also for diminishing Aβ burden, will hold up in humans receiving active tau immunizations. It is unlikely that this will be clarified in ongoing active tau immunization trials as the enrolled subjects are likely to have already substantial Aβ deposits, whose development may have plateaued because of synaptic loss. As in our study, prophylactic therapy will likely be required, which could be assessed first in individuals with genetic mutations that are known to cause AD or related tauopathies. However, because the extent of tau pathology correlates much better with cognitive deficits than Aβ burden, tau immunotherapies are likely to provide clinical benefits at later stages of AD than treatments that directly target Aβ.

## Electronic supplementary material

Supplementary Figures - Original blots
