# Supplementary material for: Prophylactic Active Tau Immunization Leads to Sustained Reduction in Both Tau and Amyloid-β Pathologies in 3xTg Mice
Source: Sci Rep. 2017 Dec 6;7:17034. doi: 10.1038/s41598-017-17313-1 (PMC5719023; doi:10.1038/s41598-017-17313-1)

SREP-17-32224A

Prophylactic Active Tau Immunization Leads to Sustained Reduction in Both Tau and Amyloid-β Pathologies in 3xTg Mice

Hameetha Rajamohamedsait[1], Suhail Rasool[1], Wajitha Rajamohamedsait[1], Yan Lin[1], Einar M. Sigurdsson[1, 2*]

Departments of [1]Neuroscience and Physiology, and [2]Psychiatry, New York University School of Medicine, 550 First Avenue, New York, NY 10016

***Correspondence:**

Einar M. Sigurdsson, Ph.D.

Department of Neuroscience and Physiology

Medical Science Building, MSB459

New York University School of Medicine, 550 First Avenue, New York, NY 10016

E-mail: einar.sigurdsson@nyumc.org

Supplementary Information

Figure 4 A-1

CP27 Soluble Tau

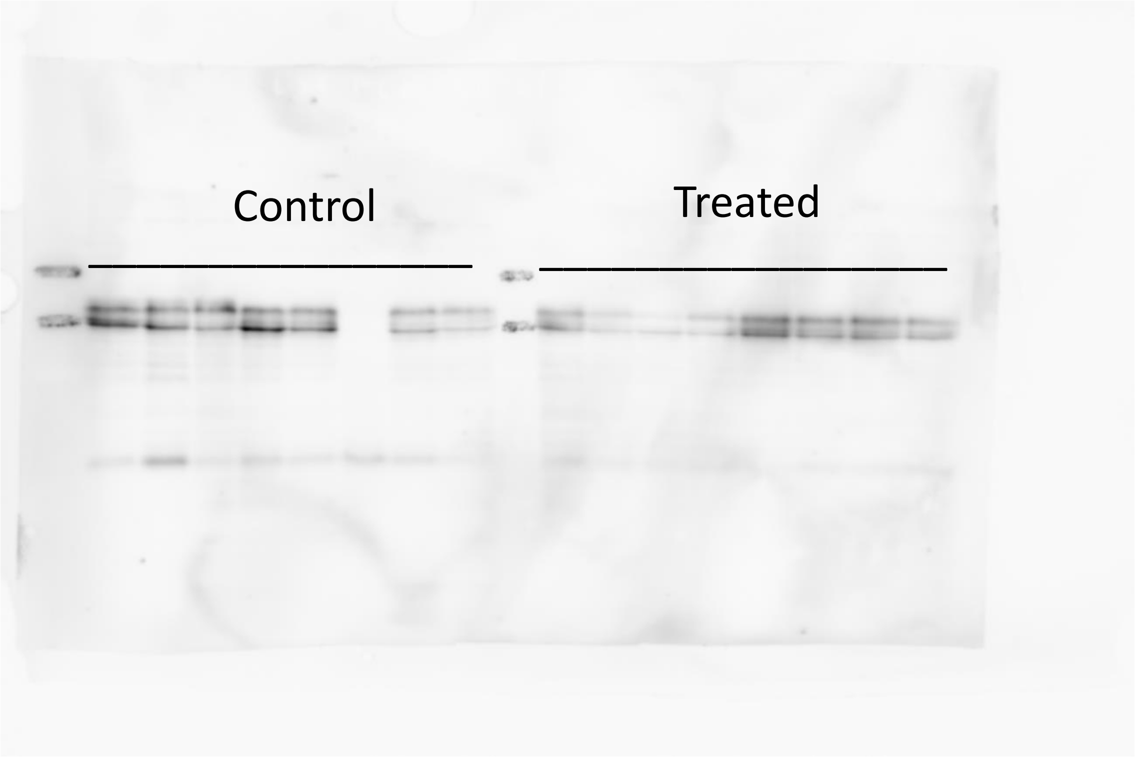

Supplementary Information

Figure 4 A-2

CP27 Insoluble Tau

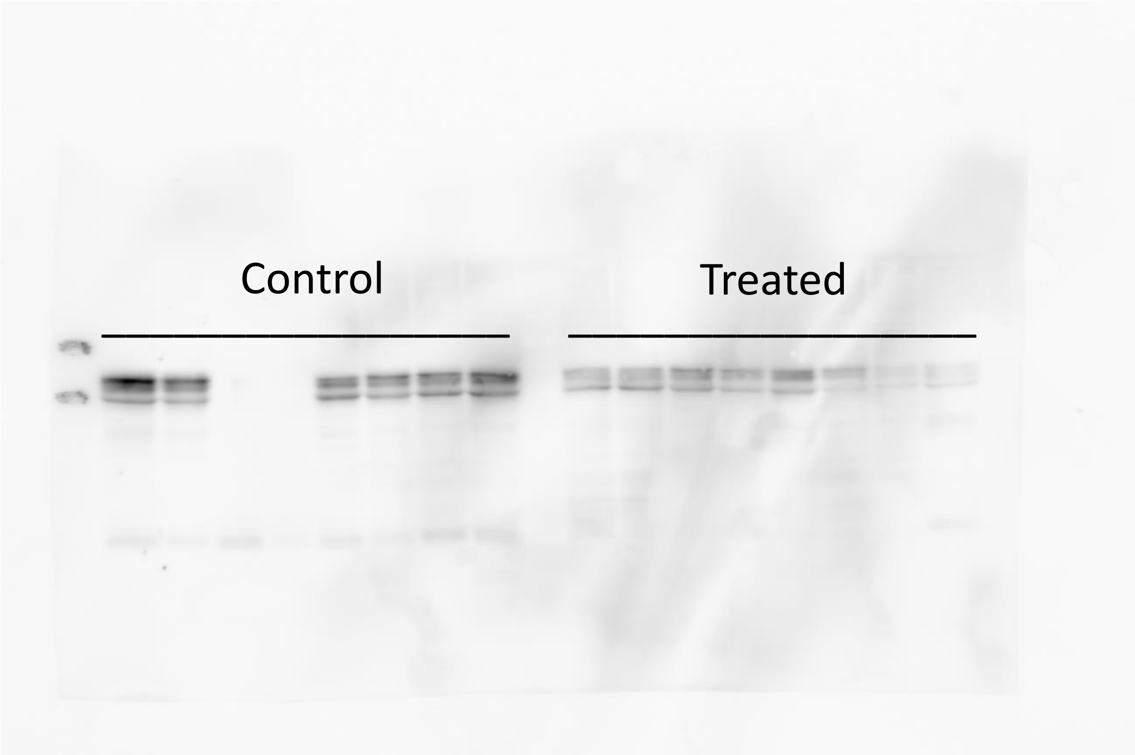

Supplementary Information

Figure 5 A-1

PHF1 Soluble Tau

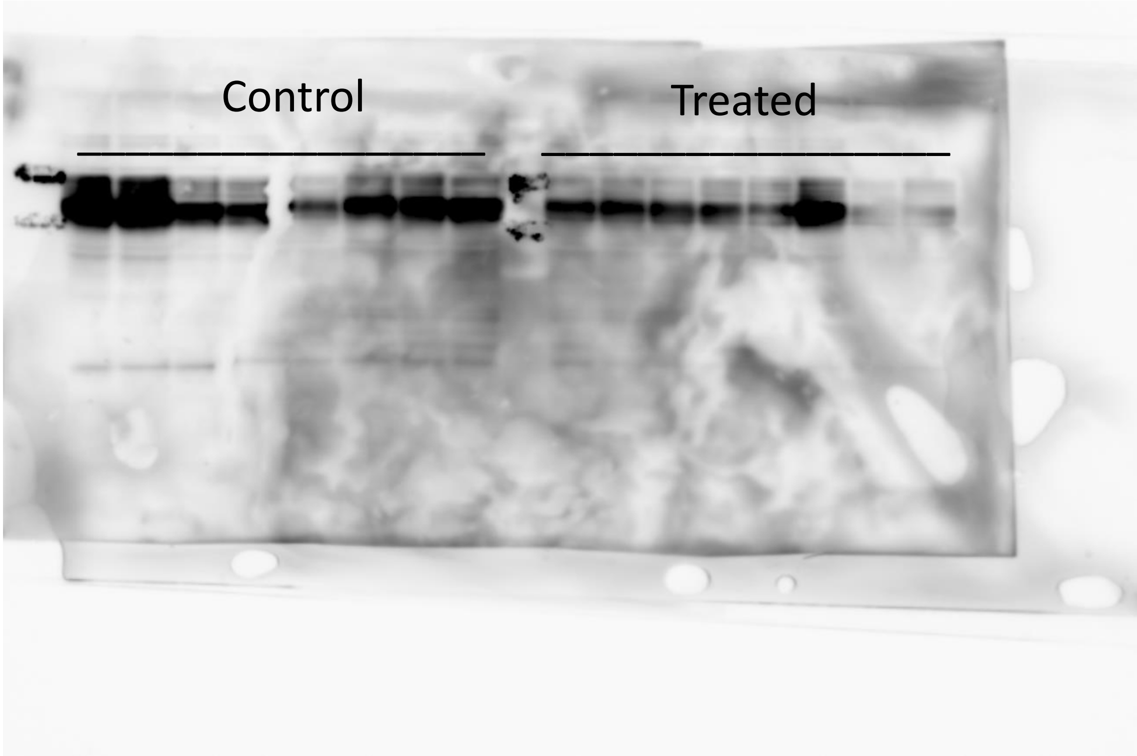

Supplementary Information

Figure 5 A-2

PHF1 Insoluble Tau

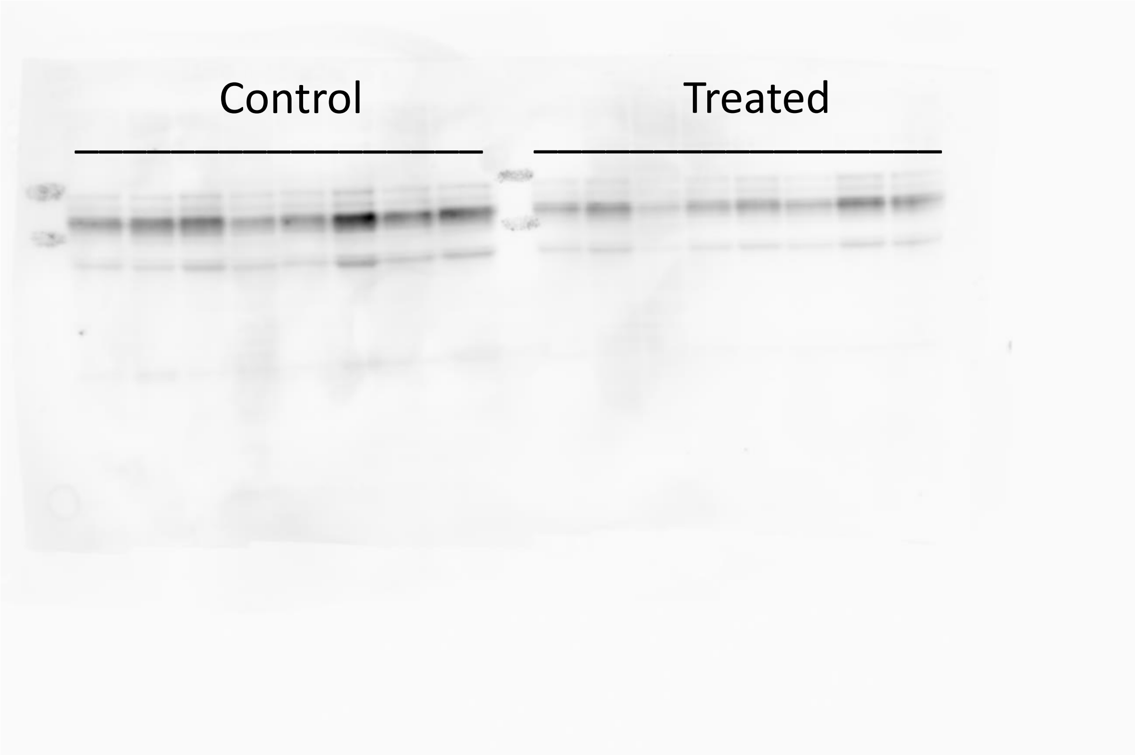

Supplement: Supplementary file 1 — Supplementary Figures - Original blots [file 41598_2017_17313_MOESM1_ESM.pdf]
